# Association of same-day urinary phenol levels and cardiac electrical alterations: analysis of the Fernald Community Cohort

**DOI:** 10.21203/rs.3.rs-4445657/v1

**Published:** 2024-05-31

**Authors:** Jack Rubinstein, Susan M. Pinney, Changchun Xie, Hong-Sheng Wang

**Affiliations:** University of Cincinnati; University of Cincinnati; University of Cincinnati; University of Cincinnati

**Keywords:** environmental chemicals, phenol, bisphenol, human population, heart electrical properties

## Abstract

**Background:**

Exposure to phenols has been linked in animal models and human populations to cardiac function alterations and cardiovascular diseases, although their effects on cardiac electrical properties in humans remains to be established. This study aimed to identify changes in electrocardiographic (ECG) parameters associated with environmental phenol exposure in adults of a midwestern large cohort known as the Fernald Community Cohort (FCC).

**Methods:**

During the day of the first comprehensive medical examination, urine samples were obtained, and electrocardiograms were recorded. Cross-sectional linear regression analyses were performed.

**Results:**

Bisphenol A (BPA) and bisphenol F (BPF) were both associated with a longer PR interval, an indication of delayed atrial-to-ventricle conduction, in females (p < 0.05) but not males. BPA combined with BPF was associated with an increase QRS duration, an indication of delayed ventricular activation, in females (P < 0.05) but not males. Higher triclocarban (TCC) level was associated with longer QTc interval, an indication of delayed ventricular repolarization, in males (P < 0.01) but not females. Body mass index (BMI) was associated with a significant increase in PR and QTc intervals and ventricular rate in females and in ventricular rate in males. In females, the combined effect of being in the top tertile for both BPA urinary concentration and BMI was an estimate of a 10% increase in PR interval. No associations were found with the other phenols.

**Conclusion:**

Higher exposure to some phenols was associated with alterations of cardiac electrical properties in a sex specific manner in the Fernald cohort. Our population-based findings correlate directly with clinically relevant parameters that are associated with known pathophysiologic cardiac conditions in humans.

## BACKGROUND

Phenols are a group of synthetic chemicals that are extensively used in consumer goods and products [1–3]. For example, bisphenol A (BPA) and its analogs bisphenol F (BPF) and bisphenol S (BPS) are used in the manufacturing of a wide range of consumer plastics goods such as food containers, food cans, water bottles, baby bottles, dental sealants and water pipes, parabens are used as preservatives in cosmetics, pharmaceuticals, foods, and beverages, and triclosan (TCS) and triclocarban (TCC) are antibacterial chemicals used in personal care products such as detergents, soaps, lotions, toothpastes, and shampoos [1, 4–6]. In addition, 2.4-dichlorophenol (2,4-DCP) is used in synthesis of phenoxy acid herbicides. Due to their high production and extensive use, phenols are common environmental chemicals, and are present in surface and drinking water, soils, household dust, air, and sediments [1, 5, 6]. There is well-documented human exposure to environmental phenols. For example, in the earliest measures in urine from the National Health and Nutrition Exam Survey (NHANES, 2003–2004), BPA was detected in 92.6% of the population with a geometric mean urinary concentration of 2.64 ng/mL (years 2003–04), 2.4-DCP in 81.2% of the population with a geometric mean of 1.04 ng/mL (years 2003–04), and TCC in 74.6% of the population with a geometric mean of 13.0 ng/mL [7–9].

Phenols are also known environmental endocrine disruptors (EDCs), which interfere with the actions of native sex hormones and have a range of potentially adverse impacts on human health [1–3]. Several phenols have been shown to have cardiovascular toxicities. In particular, multiple epidemiological studies and independent analyses of NHANES have demonstrated that higher urinary BPA concentrations were associated with cardiovascular diagnoses, including coronary heart disease, angina, and peripheral arterial disease [10–12]. Experimental studies show that BPA exposure results in alteration of cardiac Ca^2+^ handing, arrhythmogenesis, cardiac remodeling, alteration of cardiac function, oxidative stress and altered autonomic regulation [13–18]. Many of these cardiac toxicities of BPA in experimental studies are shared by its analogs, including BPS and BPF [4, 16, 19, 20]. In addition, TCC has been shown to result in cytotoxicity and arrhythmic beating in human inducible pluripotent stem cell derived cardiomyocytes (hiPSC-CMs) [21], and TCS has been shown to impair cardiac excitation-contraction coupling and cardiomyocyte differentiation [22, 23].

A notable gap in the current knowledge on the cardiac toxicity of phenols is the lack of understanding of the impact of these chemicals on the cardiac electrical properties in humans. Electrical excitation and conduction are fundamental to normal cardiac physiology [24]. Cardiac electrical abnormalities play a key role in cardiac diseases and are a major contributor to human morbidity and mortality [25–27]. Experimental evidence shows that BPA and its analogs can impact several cardiac ion channels including the L-type and T-type Ca^2+^ channels, the rapid delay rectifier K^+^ channel and ryanodine receptors, as well as affecting electrophysiological properties in hiPSC-CMs, canine ventricular myocytes, and rodent cardiac myocytes [15, 16, 19, 28–30]. Such bisphenol-induced electrical alterations can result in the development of arrhythmias under certain pathophysiological conditions in hiPSC-CMs and rodent hearts [16, 18, 31]. These findings point to the need to define the impact of environmental phenols on the cardiac electrical properties of human hearts.

In the present cross-sectional study, we aimed to identify changes in electrocardiographic (ECG) parameters in a midwestern large population, known as the Fernald Community Cohort (FCC). We characterized urinary phenol concentrations in members of the FCC and examined the association of phenol exposure and alterations of cardiac electrical properties, using key ECG parameters from examinations done on the same day as the urine collection.

The FCC consists of nearly ten thousand individuals who participated in medical monitoring because they had lived for at least three years within five miles of a uranium processing plant. The facility’s primary mission was to produce high-purity uranium metal necessary for nuclear weapons production. A uranium exposure algorithm was developed by the Centers for Disease Control and Prevention (CDC) as part of the Fernald Dosimetry Reconstruction project [32]. A significant proportion of those in the screening program received airborne environmental uranium exposure that was negligible compared to background exposure [33] and were categorized into the lowest exposure group, with exposure ≤ 0.2 μg/m^3^-yr beyond background. Only those in this lowest exposure category were eligible for the present study. The FCC cohort, with its ECG data and concurrently collected biosamples at the time of the first examination, offered an invaluable dataset for examining the potential cardiac electrical impact of phenols in humans. This study addressed the hypothesis that in humans, higher exposure level to at least some environmental phenols is associated with changes in ECG markers of cardiac function and risk of arrhythmia. The key ECG markers used in this study included QT interval, PR interval, QRS duration and RR interval (or heart rate). QT interval represents the duration of ventricular excitation of the electric cardiac cycle. PR interval represents atrium-to-ventricle conduction through the atrioventricular (AV) node which is modulated by both the AV node conduction properties as well as vagal tone and aging. QRS duration represents ventricular depolarization. Lastly, RR interval represents the time elapsed between two successive ventricular beats and is the reciprocal of heart rate.

## METHODS

### Urine sample storage, subject selection and ECG analysis

Fernald Cohort urine samples and ECG data were collected at the first examination in 1991–1994. Urine samples were aliquoted and immediately stored, without buffer, in 1.0 ml polypropylene tubes at −80°C. Members of the Fernald cohort eligible for the present study were from the portion of the cohort with negligible uranium exposure, were 18–80 years of age at the time of the first examination. Those taking beta-blockers, antiarrhythmic drugs, or had known cardiac conduction abnormalities, the long QT syndrome, or cardiomyopathy were not eligible. Of the remaining members of the cohort, 302 males and 302 females were randomly selected. The mean age was 39 ± 13 years. The vast majority self-identified as white with a broad representation of socio-economic and educational backgrounds (Table 1). All had interpretable ECG values.

ECGs were read by physicians who were board certified in cardiology. Four key ECG parameters, corrected QT interval, PR interval, duration of the QRS complex, and RR interval were included in this analysis. QT interval was corrected for heart rate (QTc). Descriptive statistics were used to identify possible outlying observations, and the tenability of parametric assumptions were assessed prior to estimating the models to evaluate our primary hypotheses.

### Measurement of urine phenol concentrations

Twelve phenol analytes were analyzed in the urine (ng/mL) of 604 participants. The Centers for Disease Prevention and Control (CDC) Division of Laboratory Sciences conducted the analysis using an automated on-line column switching HPLC-MS/MS method with peak focusing. If the percentage of detection in the population was less than 40% then analysis was not performed on the analyte[8]. Phenols analyzed for this study included BPA, benzophenone-3 (BP-3), bisphenol-F (BPF), 2,4, dichlorophenol (24-DCP), 2,5 dichlorophenol (25-DCP), methyl paraben (M-PB), propyl paraben (P-PB), triclocarban (TCC), and triclosan (TCS).

### Statistical analysis

Median and mean statistics for the outcomes and risk factors were calculated for both the entire cohort and stratified by sex. The sex specific statistics were tested for significance using Fisher’s exact test for the association of the phenol urinary biomarker level with sex for categorical variables and a linear model was used to test the association with sex for continuous variables. P values were provided based on F test in the Type III Sum of Squares output. To determine the association between the urinary concentrations of phenols and alteration of ECG parameters, creatinine-corrected urine phenol concentrations (BPA, DCP24, DCP25, BPF, BP3, MPB, PPB, TCS, TCC, combination of BPA and BPF, combination of DCP24 and DCP25) were log-transformed or categorized into tertiles for analyses. Linear regression models were used, where the ECG parameter in miliseconds (ms) was dependent variable and creatinine-corrected environmental chemical was independent variable. Because of the sex differences in ECG parameters and since phenols are known endocrine disruptors, sex-specific analyses were conducted in order to fully characterize the effect of creatinine-corrected environmental phenols on ECG parameters for each gender separately.

Different ECG parameters had different sets of covariates but all included sex, age at exam, smoking pack-year history exam, alcohol consumption at enrollment, and physician(s) reading the ECG (to account for possible physician-specific variations in ECG interpretation), using the F test in the Type III Sum of Squares output. Other parameters such as serum cholesterol (mg/dL) and triglyceride (mg/dL) were included after a review of the literature and retained as covariates in all final analyses because of their statistical significance except for the effect on QRS duration. Serum potassium concentration was included in the analysis of QTc as reported in the literature [34]. The use of oral contraceptives or hormone replacement therapy (yes/no) were included in analyses of QTc and ventricular rate in the female subcohort.

## RESULTS

As shown in Table 1a, the median and mean values for the ECG parameters of the participants were within normal limits. There was moderate use of alcohol and smoking among this cohort. The measured serum triglycerides and cholesterol values are presented and are within physiological range.

There were notable differences in the ECG parameters between male and female subjects at baseline (Table 1b). Specifically, PR interval and QRS duration were all significantly longer in males, while QTc was significantly longer and heart rate significantly faster in females. There was no significant sex difference in age, BMI or smoking though females had significantly lower triglyceride, cholesterol and serum K^+^ levels. Demographic information is presented by sex in Table 1c showing a broad socio-economic distribution without significant differences between males and females. The participants are predominantly white which reflects the typical racial and ethnic composition of these rural Southwestern Ohio communities.

Urinary concentrations of the measured phenols stratified by sex are shown in Table 2. The cohort was widely exposed to the tested phenols with the exception of BPS, a newer BPA substitute. BPA was detected in 99.3% of the cohort with a mean concentration of 2.3 and 2.4 ng/mL (95% CI 2.1 to 2.6, and 2.2 to 2.5), in females and males, respectively. These BPA exposure levels in urines collected in 1990 to 1993 are similar to later reported values in the US populations [8, 35, 36]. Key differences between the sexes include significantly higher levels of BP3, M-PB and P-PB in females, and higher levels of DCP24, TCC and TCS in males. Levels of DCP25, BPA, BPF and BPS were similar between males and females.

The sex specific associations between BPA exposure and covariates and the measured ECG parameters are shown in Table 3. In females but not males, higher urinary BPA was associated with longer PR interval (4.01 ms increase in PR interval per unit increase in ln[BPA]; P < 0.05). In addition, higher BMI in females was associated with longer QTc (0.40 ms per unit BMI; P < 0.05), longer PR interval (0.59 ms per unit BMI; P < 0.01), and longer RR interval (i.e., slower heart rate; 0.36 ms in RR per unit BMI; P < 0.01). In males, BMI was only associated with longer RR interval (0.49 ms per unit BMI; P < 0.01). In females, age was associated with a small reduction of QRS (0.09 ms per year; P < 0.05), and in males, age was associated with longer QTC (0.26 ms per year; P < 0.01) and PR interval (0.51 ms per year; P < 0.001).

Further, as noted above, sex was a strong covariate; the female sex was associated with longer QTc (7.28 ± 1.73 ms; P < 0.0001), shorter PR interval (8.44 ± 1.92 ms; P < 0.0001), shorter QRS (8.94 ± 0.89 ms; P < 0.0001), and higher RR interval (4.63 ± 1.11 ms; P < 0.0001).

The associations between other phenols and changes in ECG parameters are found in Table 4. Similar to BPA, higher urinary BPF and BPA plus BPF were associated with longer PR interval in the female population (3.4 and 5.09 ms increase in PR interval per unit increase in ln[BPF] and ln[BPA + BPF], respectively; P < 0.01). In females, higher BPA plus BPF was associated with longer QRS duration (1.5 ms increase in QRS per unit increase in ln[BPA + BPF]; P < 0.05). In males, TCC exposure was associated with prolongation of QTc (1.38 ms per unit of ln[TCC]; P < 0.01). When females and males were analyzed as a single cohort there were only significant associations noted between QTc and TCC and PR interval and BPA + BPF (Suppl Table 1). No significant associations were noted between any phenol and RR interval.

**Figure 1:**
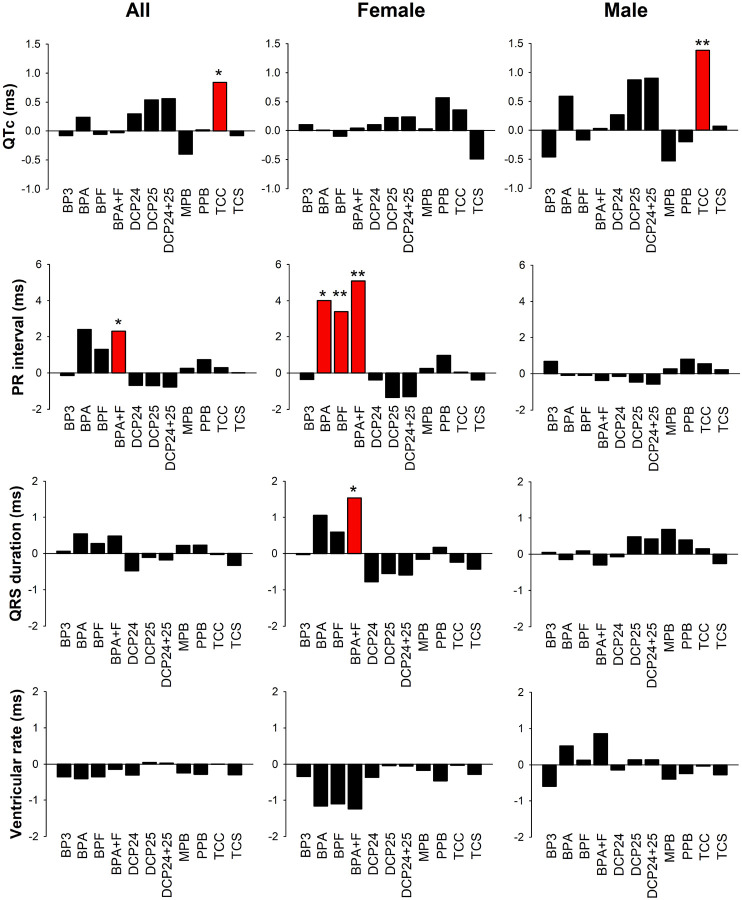
Graphic summary of the effect of phenol exposure on ECG parameters in the Fernald Cohort. Effect size represents the change in the value of ECG parameters with a change in one unit of log transformed urinary phenol concentration. Red bars illustrate effects that have statistical significance. *: P < 0.05; **: P < 0.01.

The association between phenols and ECG parameters is summarized in Fig. 1. The strong sex dichotomy is evident. The female cohort showed significant increases in PR interval associated with BPA, BPF, and BPA + BPF exposure, while BPA + BPF exposure was also associated with increased QRS duration. The male cohort demonstrated an association between TCC levels and QTC, but no other associations were noted.

**Figure 2:**
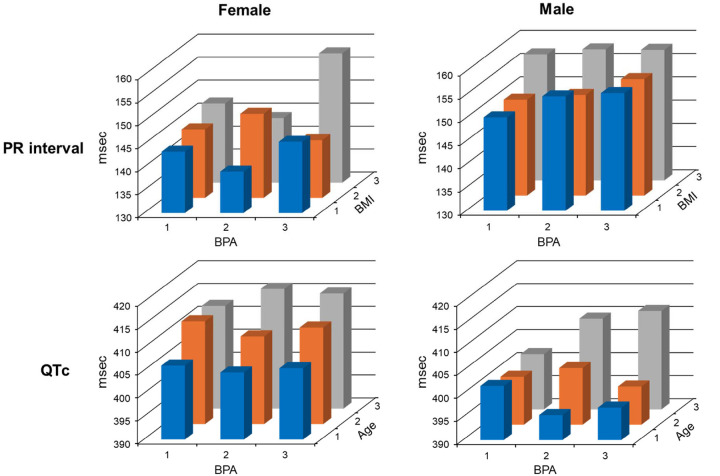
Top: Relationship between PR interval, urinary BPA concentration, and BMI, with both urinary BPA and BMI divided into tertiles. BPA categories: 1, < 1.65; 2, 1.65 – 2.96; 3, > 2.96 (ng/mL); BMI categories: 1, < 24.2; 2, 24.2 – 28.4; 3, > 28.4. Bottom: Relationship between QTc, urinary BPA concentration, and age, with both urinary BPA and age divided into tertiles. BPA categories: 1, < 1.65; 2, 1.65 – 2.96; 3, > 2.96 (ng/mL); age categories: 1, 18 – 31; 2, 32 – 45; 3, 46 – 77 (years old).

While higher phenol exposure was only associated with small changes in ECG parameters, such change may be more pronounced in certain BMI or age subpopulation groups. Higher BPA was associated with PR interval prolongation in females (see Table 3 and Fig. 1). Dividing both urinary BPA and BMI into tertiles revealed such association was particularly pronounced in females in the top BMI tertile group (Fig. 2, top). In females, the combined effect of being in the top tertile for both BPA urinary concentration and BMI was an estimate of 14.8 ms, or about 10% increase in PR interval (Fig. 2, top). Similarly, dividing the cohort into tertiles based on urinary BPA and age suggests that BPA may have an association with QT prolongation in the older age group. Particularly in males, QTc interval increased with BPA exposure in the older age group but not in the mid to lower age groups (Fig. 2, bottom).

## DISCUSSION

Extensive experimental and epidemiological evidence points to the cardiac toxicity of bisphenol-type environmental chemicals. However, the impact of bisphenols and other phenols on the cardiac electrical properties in humans is entirely unknown. This is the first study to examine the associate of phenol exposure and ECG alterations in a human cohort, and provides insights into phenol associated, sex-specific changes in cardiac electrical properties. The key findings of this study relate to the changes observed in ECG parameters, PR interval and QTc in particular, as it pertains to BPA and other phenol levels when analyzed by sex. These alterations of cardiac electrical properties have clear physiological and pathological implications.

PR interval represents the electrical conduction between the atria and the ventricle, and prolongation of the interval is associated with increasing age as the AV node function decreases [37, 38]. This is relevant as advanced AV blocks are a common indication for placement of artificial cardiac pacemakers [39] and are also relevant in an aging population that is prone to develop atrial arrhythmias (such as atrial fibrillation) that depend on normal AV conduction to maintain cardiac output [40–42]. In this study we found the PR interval increases with age in both males and females as expected, and increases in females in association with BPA and BPF exposure. Further, the combined effect of BPA exposure with elevated BMI was associated with an approximate 10% increase in PR interval in females. This association between PR prolongation and bisphenol exposure is generally consistent with published experimental data showing that exposure to BPA delayed AV conduction and increased the PR interval in excised female rat hearts [43]. Conduction velocity in cardiac tissue is determined by the size of the action potential-generating inward current [44]. It is possible that BPA reduces AV node conduction through inhibition of the L-type Ca^2+^ current, which is the dominant inward current in AV node myocytes and a key determinant of AV node conduction velocity [44, 45]. Recently, we have shown that 1 nM BPA acutely inhibited the L-type Ca^2+^ current in hiPSC-CMs, including in nodal-like myocytes [16], and that long term exposure to 1 nM BPA inhibited the L-type Ca current in hiPSC-CMs through reduction of the L-type Ca^2+^ channel protein expression level [19]. Similarly, low-doses of BPA inhibited the L-type Ca^2+^ current in female rat cardiomyocytes in a dose-dependent manner [14]. At higher, μM doses, BPA has also been shown to suppress the L-type current/channel in hiPSC-CMs and heterologously expressed in HEK cells [29, 30]. Such BPA-mediated inhibition of the L-type Ca^2+^ channel may provide a cellular mechanism for the observed prolongation of the PR interval in the Fernald Cohort.

It has been well-established that prolongation of the QT interval is a key arrhythmogenic mechanism and are associated with adverse cardiac outcomes, specifically sudden cardiac death [25–27]. QT prolongation is an indication of delay in ventricular repolarization, which can lead to ectopic ventricular excitation and malignant ventricular arrhythmias [25, 26, 46]. QT prolongation is often caused by genetic mutations in cardiac ion channels, predominantly K^+^ and Na^+^ channels, and the resulting prolongation of ventricular action potential [47, 48]. QT prolongation can also be produced by adverse blockade of cardiac ion channels by drugs or environmental chemicals, and is one of the central issues in cardiac safety and cardiotoxicity assessment [49–53]. Examples of QT prolongation-related cardiac toxicity of environmental chemicals include trivalent arsenic As^3+^ and organophosphate poisoning [54–57]. Here, we show that higher urine level of TCC was associated with longer QTc in males but not females. TCC, an antibacterial chemical once common in personal care products, is now banned for usage in the US. Information on the association between TCC exposure and QT prolongation toxicity may help retrospective understanding of the health outcomes of the Fernald Cohort. In addition, even though there is no association between BPA exposure and QT change at the cohort level, we found that in older males, higher BPA exposure may have an association with QTc prolongation. While QTc difference between the lowest to the highest groups was modest (about 20 msec), from a population perspective even smaller increases in QTc have been shown to be associated with increased risk of adverse outcomes. For instance, Zhang and colleagues found that any increase in QT interval was associated with a small but measurable change in total and cardiovascular mortality and specifically sudden cardiac death [58]. Our findings have important implications for the QT prolongation cardiotoxicity of BPA, and likely other related bisphenol-type chemicals, in older males.

From a cellular physiologic perspective, BPA has been shown to inhibit the IKr K current, which plays a key role in cardiac repolarization. We showed in our previous studies that acute exposure to environmentally relevant low dose BPA (1 nM) inhibited the IKr current in hiPSC-CMs and canine ventricular myocytes. Such IKr inhibition resulted in delay in action potential repolarization in hiPSC-CMs and canine ventricular myocytes, and prolongation of QT interval in canine ventricular tissue [15, 16]. The pro-arrhythmic toxicity of such BPA-induced repolarization delay was illustrated by the marked increase in arrhythmic events under BPA exposure combined with pathophysiological conditions [16]. In addition, acute exposure to high doses (mid to high μM) BPA has also been shown to inhibit IKr in hiPSC-CMs and hERG (the molecular correlate of human IKr current) expressed in HEK 293 [30]. While these acute experimental exposure findings cannot be directly extrapolated to the exposure scenario in humans, they nevertheless offer a possible cellular mechanism of the observed QT prolongation associated with bisphenol exposure in the cohort.

No change in QRS duration or heart rate was found to be associated with exposure to any single tested phenol, while higher urine BPA plus BPF level was associated with slight QTS prolongation in females. QRS duration represents depolarization of the ventricles and is prolonged under disease conditions including heart failure, prior infarction, and/or development of a bundle branch block. The etiology of the QRS prolongation associated with BPA plus BPF levels is unknown, and further experimental and human population studies are needed to fully clarify this. That being noted, Hnatkova and colleagues found that, in healthy individuals, QRS duration is slightly longer in males in comparison with females (103.2 msec vs 98.7 msec) [59], which is similar to the small differences we observed in this study.

The lack of effect of phenols on heart rate in the Fernald participants contrasts with previously reported acute stimulatory effect of bisphenols on ex vivo rodent heart rate and pacing rate of nodal hiPSC-CMs [16, 30, 31], and likely reflects the difference between chronic and acute phenol exposures. Further, the human heart rate is controlled by cardiac factors (such as sinus node automaticity) as well as extracardiac factors such as vagal tone, adrenaline secretion and sympathetic activity. The autonomic control of the heart rate in vivo may be another reason for the contrasting cohort vs experimental findings on this parameter.

We show here that higher exposure to some phenols was associated with alternations of ECG properties in the Fernald Cohort. Individual persons may experience more or less of an effect of BPA exposure on the ECG properties due to individual differences in absorption and metabolism of BPA. Regardless, it should be noted that these ECG changes at the population level are moderate and generally within the normal ECG parameter ranges. While these changes alone are unlikely to result in clinically significant cardiac electrical disease in healthy individuals, we believe that higher phenol exposure is a risk factor that can contribute to cardiac electrical abnormalities, particularly in subpopulations with existing pathophysiological conditions and predisposition. One such scenario is the combined effect of bisphenol-associated PR interval prolongation and BMI in females. As shown in Fig. 2, high exposure combined with high BMI can lead to sizable PR alteration in females. It is also possible that bisphenol-associated PR prolongation, particularly in high exposure individuals, can exacerbate existing AV block in patients with heart conduction disorders. Also, as noted above, subsetting the cohort into tertiles based on age reveals clue on the possible QT prolongation toxicity of BPA in the older subpopulation. Such a subpopulation-based toxicity assessment approach can better identify cardiac toxicities of environmental chemicals that would otherwise be difficult to identify in the general population.

The cross-sectional nature of the study allows us to determine association but not causality. Further, the temporal variability of BPA, and likely other phenols, is well known [60–63], and a one-time measurement of phenol levels does not represent lifetime exposure. However, even with these limitations, the FCC is highly valuable because it is unusual to have a large population-based cohort with simultaneous urine specimen collection and ECG recordings, which allowed us to determine the association of real-time (not lifetime) phenol exposure with ECG markers.

In conclusion, our population-based study shows, for the first time, that higher urinary levels of environmental phenols, including BPA, BPF, and TCC, were associated with sex-specific alterations in cardiac electrical-conduction properties in the Fernald Cohort. Our findings correlate with clinically relevant parameters that are associated with known pathologic conditions in humans. Our findings may have implication for the cardiac toxicity of these chemicals, particularly in predisposed subpopulations.

## Figures and Tables

**Table 1a:** Electrocardiograph and other basic medical information for the Fernald Community Cohort.

	N	Median	Mean	Std	Min	Max	Q1	Q3
Ventricular rate (bpm)	604	67.0	67.8	10.9	40.0	109.0	60.5	74.0
PR interval (ms)	604	148.0	150.3	21.0	100.0	208.0	136.0	164.0
QRS duration (ms)	604	88.0	91.5	10.5	60.0	156.0	84.0	96.0
Corrected QT (QTc) (ms)	604	404.5	406.0	17.0	280.0	458.0	396.0	416.0
Alcohol at enrollment (drinks/wk) (for those who drink)	173	4.0	7.3	9.1	1.0	66.0	2.0	10.0
Smoking pack years at enrollment (for those who smoke)	154	0.8	0.9	0.4	0.1	2.5	0.5	1.0
Age (yr)	604	39.7	39.6	13.4	18.0	76.7	29.2	49.0
BMI	604	26.0	27.2	5.5	16.6	51.7	23.4	30.0
Triglyceride (mg/dL)	601	99.0	126.1	101.6	15.0	1270.0	64.0	159.0
Cholesterol (mg/dL)	601	195.0	196.4	40.7	103.0	332.0	168.0	221.0
Potassium (mEq/L)	602	4.5	4.5	0.4	3.4	6.1	4.2	4.8

bpm: beats per minute.

MBI: body mass index.

**Table 1c:** Demographic information.

	Overall			Females			Males		
	total	n	%	total	n	%	total	n	%
Race: White	604	602	99.7%	302	300	99.3%	302	302	100%
Race: American Indian	604	2	0.3%	302	2	0.7%	302	0	0.0%
Educate: High school graduate or less	604	281	46.5%	302	143	47.4%	302	138	45.7%
Educate: Some college or Vocational training	604	204	33.8%	302	102	33.8%	302	102	33.8%
Educate: College graduate	604	78	12.9%	302	38	12.6%	302	40	13.2%
Educate: Postgraduate or professional degree	604	41	6.8%	302	19	6.3%	302	22	7.3%
Income in 1990–1994: Less than $20,000	574	119	20.7%	288	62	21.5%	286	57	19.9%
Income in 1990–1994: $20,000 – $49,999	574	303	52.8%	288	156	54.2%	286	147	51.4%
Income in 1990–1994: $50,000 - or more	574	152	26.5%	288	70	24.3%	286	82	28.7%
Oral contraception				302	47	15.60%			
Hormone replacement				302	32	10.60%			

**Table 2:** Urinary phenol measurements in the Fenald Community Cohort stratified by sex.

	FEMALES							
Phenol (ng/mL)	N	Median	Median 95% CI	Geometric Mean	Geometric Mean 95% CI			
BP3	302	9.2	(6.6–11.3)	11.8	(9.0–15.3)			
BPA	302	2.5	(2.1–2.8)	2.3	(2.1–2.6)			
BPF	302	0.3	(0.2–0.4)	0.4	(0.3–0.5)			
BPS	302	0.07	(0.07–0.07)	0.11	(0.08–0.14)			
DCP24	302	1.1	(1.0–1.2)	1.2	(1.0–1.3)			
DCP25	302	16.6	(13.2–21.1)	20.2	(16.9–24.2)			
M-PB	302	178.1	(133.1–225.5)	142.8	(120.6–169.1)			
P-PB	302	69.2	(54.4–92.6)	48.4	(39.9–60.3)			
TCC	302	1.85	(1.2–3.7)	2.5	(1.9–3.2)			
TCS	302	2.95	(2.4–3.7)	5.7	(4.7–7.0)			
	MALES					Overall		
Phenol (ng/mL)	N	Median	Median 95% CI	Geometric Mean	Geometric Mean 95% CI	LOD (ng/mL)	%>LOD	Female vs male P-value
BP3	302	3.05	(2.5–3.8)	3.8	(3.1–4.7)	0.4	92.2%	<0.0001
BPA	302	2.5	(2.2–2.8)	2.4	(2.2–2.5)	0.2	99.3%	0.5489
BPF	302	0.4	(0.3–0.5)	0.6	(0.5–0.7)	0.2	63.1%	0.0056
BPS	302	0.07	(0.071–0.071)	0.14	(0.09–0.20)	0.01	18.2%	0.4362
DCP24	302	1.9	(1.6–2.2)	1.9	(1.6–2.1)	0.1	99.2%	<0.0001
DCP25	302	24.6	(18.3–19.9)	25.8	(21.8–31.4)	0.1	100.0%	0.0518
M-PB	302	20.4	(16.1–28.4)	24.9	(21.0–29.7)	1	99.8%	<0.0001
P-PB	302	3	(2.6–4.4)	3.9	(3.1–4.9)	0.1	99.3%	<0.0001
TCC	302	6.9	(4.1–12.0)	5.5	(4.2–7.2)	0.1	93.0%	0.0005
TCS	302	12	(5.5–18.4)	16.9	(12.9–22.2)	1.7	67.4%	<0.0001

LOD: limit of detection.

**Table 3:** Effect of BPA exposure on ECG parameters.

FEMALES												
	QTc (ms)			PR interval (ms)			QRS duration(ms)			RR interval (ms)		
	β	SE	P value	β	SE	P value	β	SE	P value	β	SE	P value
BPA (ln[BPA])	0.005	1.354	1.00	4.015	1.767	**<0.05**	1.063	0.675	0.12	−1.167	1.000	0.24
Age (year)	0.152	0.100	0.13	0.221	0.117	0.06	−0.092	0.040	**<0.05**	−0.055	0.074	0.46
BMI	0.403	0.174	**<0.05**	0.587	0.223	**<0.01**	0.118	0.082	0.15	0.359	0.127	**<0.01**
Smoke (CPY)	3.169	2.631	0.23	1.635	3.405	0.63	−0.671	1.281	0.60	−2.104	1.949	0.28
Alcohol (drinks/wk)	0.003	0.320	0.99	−0.519	0.411	0.21	0.032	0.156	0.84	0.395	0.233	0.09
Cholesterol (mg/dL)	0.070	0.032	**<0.05**	−0.020	0.042	0.63				0.020	0.024	0.41
Triglyceride (mg/dL)	0.012	0.015	0.43	0.027	0.020	0.18				0.012	0.011	0.28
Oral contraception	1.498	3.065	0.63							1.783	2.267	0.43
Hormone replacement	2.158	3.365	0.52							−0.331	2.486	0.89
Potassium (mEq/L)	−4.530	2.470	0.07									
Physician			0.58			0.17			0.78			0.46
MALES												
	QTc (ms)			PR interval (ms)			QRS duration(ms)			RR interval (ms)		
	β	SE	P value	β	SE	P value	β	SE	P value	β	SE	P value
BPA (ln[BPA])	0.593	1.749	0.74	−0.094	1.873	0.96	−0.146	1.046	0.89	0.518	0.974	0.60
Age (year)	0.257	0.095	**<0.01**	0.511	0.102	**<0.001**	−0.065	0.052	0.21	0.033	0.053	0.54
BMI	−0.232	0.263	0.38	0.008	0.285	0.98	−0.116	0.153	0.45	0.489	0.148	**<0.01**
Smoke (CPY)	3.360	2.690	0.21	1.087	2.908	0.71	1.976	1.618	0.22	2.140	1.512	0.16
Alcohol (drinks/wk)	0.110	0.141	0.43	−0.032	0.153	0.83	−0.109	0.085	0.20	0.119	0.079	0.14
Cholesterol (mg/dL)	−0.017	0.036	0.63	0.045	0.038	0.24				−0.002	0.020	0.91
Triglyceride (mg/dL)	0.021	0.010	**<0.05**	0.014	0.011	0.20				0.013	0.006	**<0.05**
Potassium (mEq/L)	−4.100	3.170	0.20									
Physician			0.32			0.19			0.55			0.95

QTc: corrected QT interval.

BPA: one unit of log transformed BPA concentration (ln[BPA]).

β represents the change in the length of the ECG parameter with a change in one unit of log transformed BPA concentration.

SE: standard error.

BMI: body mass index.

CPY: carton per year.

**Table 4:** Effect of phenol exposure on ECG parameters.

FEMALES												
	QTc (ms)			PR interval (ms)			QRS duration(ms)			RR interval (ms)		
Phenol	β	SE	P value	β	SE	P value	β	SE	P value	β	SE	P value
BP3	0.105	0.479	0.83	−0.364	0.632	0.57	−0.035	0.241	0.89	−0.347	0.349	0.32
BPF	−0.099	0.903	0.91	3.401	1.131	**<0.01**	0.589	0.443	0.19	−1.129	0.672	0.09
BPA + BPF	0.044	1.282	0.97	5.088	1.654	**<0.01**	1.536	0.630	**<0.05**	−1.239	0.943	0.19
DCP24	0.096	0.839	0.91	−1.378	1.103	0.21	−0.778	0.417	0.06	−0.363	0.622	0.56
DCP25	0.233	0.653	0.72	−1.354	0.860	0.12	−0.553	0.326	0.09	−0.040	0.485	0.93
DCP24 + 25	0.235	0.667	0.72	−1.318	0.879	0.14	−0.585	0.333	0.08	−0.048	0.496	0.92
M-PB	0.031	0.737	0.97	0.248	0.971	0.80	−0.164	0.367	0.65	−0.174	0.547	0.75
P-PB	0.573	0.550	0.30	0.967	0.726	0.18	0.169	0.276	0.54	−0.470	0.408	0.25
TCC	0.365	0.484	0.45	0.048	0.641	0.94	−0.238	0.247	0.34	−0.027	0.362	0.94
TCS	−0.494	0.497	0.32	−0.378	0.641	0.56	−0.433	0.247	0.08	−0.283	0.365	0.44
MALES												
	QTc (ms)			PR interval (ms)			QRS duration(ms)			RR interval (ms)		
Phenol	β	SE	P value	β	SE	P value	β	SE	P value	β	SE	P value
BP3	−0.466	0.678	0.49	0.686	0.722	0.34	0.053	0.407	0.90	−0.628	0.377	0.10
BPF	−0.173	0.799	0.83	−0.090	0.876	0.92	0.086	0.475	0.86	0.127	0.428	0.77
BPA + BPF	0.034	1.339	0.98	−0.368	1.442	0.80	−0.296	0.804	0.71	0.856	0.748	0.25
DCP24	0.269	1.086	0.80	−0.146	1.176	0.90	−0.072	0.657	0.91	−0.141	0.612	0.82
DCP25	0.874	0.938	0.35	−0.461	1.018	0.65	0.484	0.567	0.39	0.137	0.530	0.80
DCP24 + 25	0.911	0.984	0.36	−0.572	1.067	0.59	0.416	0.596	0.49	0.136	0.556	0.81
M-PB	−0.529	0.817	0.52	0.269	0.885	0.76	0.680	0.493	0.17	−0.403	0.460	0.38
P-PB	−0.206	0.651	0.75	0.802	0.701	0.25	0.395	0.392	0.32	−0.246	0.366	0.50
TCC	1.384	0.515	**<0.01**	0.548	0.564	0.33	0.153	0.314	0.63	0.036	0.292	0.90
TCS	0.070	0.451	0.88	0.218	0.492	0.66	−0.263	0.272	0.33	−0.274	0.250	0.27

QTc: corrected QT interval.

b: change in the length of the ECG parameter with a change in one unit of log transformed urinary phenol concentration.

SE: standard error.

Covariates are the same as those included in the BPA analysis as listed in Table 3.

## Data Availability

Data supporting the results reported in the article are available from the corresponding author upon request after publication.
